# Encapsulation of Naproxen in Lipid-Based Matrix Microspheres: Characterization and Release Kinetics

**DOI:** 10.4103/0975-1483.80293

**Published:** 2011

**Authors:** PK Bhoyar, DO Morani, DM Biyani, MJ Umekar, JG Mahure, YM Amgaonkar

**Affiliations:** *S.K.B. College of Pharmacy, New Kamptee, Nagpur, Maharashtra, India*

**Keywords:** Carnauba wax, hydrogenated castor oil, lipid microspheres, modified melt dispersion technique, naproxen, release kinetics

## Abstract

The objective of this study was to microencapsulate the anti-inflammatory drug (naproxen) to provide controlled release and minimizing or eliminating local side effect by avoiding the drug release in the upper gastrointestinal track. Naproxen was microencapsulated with lipid-like carnauba wax, hydrogenated castor oil using modified melt dispersion (modified congealable disperse phase encapsulation) technique. Effect of various formulation and process variables such as drug-lipid ratio, concentration of modifier, concentration of dispersant, stirring speed, stirring time, temperature of external phase, on evaluatory parameters such as size, entrapment efficiency, and *in vitro* release of naproxen were studied. The microspheres were characterized for particle size, scanning electron microscopy (SEM), FT-IR spectroscopy, drug entrapment efficiency, *in vitro* release studies, for *in vitro* release kinetics. The shape of microspheres was found to be spherical by SEM. The drug entrapment efficiency of various batches of microspheres was found to be ranging from 60 to 90 %w/w. *In vitro* drug release studies were carried out up to 24 h in pH 7.4 phosphate buffer showing 50-65% drug release. *In vitro* drug release from all the batches showed better fitting with the Korsmeyer-Peppas model, indicating the possible mechanism of drug release to be by diffusion and erosion of the lipid matrix.

## INTRODUCTION

Microspheres are defined as homogeneous, monolithic particles in the size range of about 0.1-1000 *μ*m and are widely used as drug carriers for controlled release. These systems have significant importance in biomedical applications. Microspheres can be produced for protection of core material, reduction of gastric irritation decrease in volatility, conversion of liquid to pseudo-solid, cell microencapsulation, and designing pulsatile drug delivery systems. Administration of the drug in the form of microspheres usually improves the treatment by providing the localization of the active substances at the site of action and by prolonging release of drugs.[1]

Naproxen is a newer nonsteroidal anti-inflammatory drug (NSAID), most useful drug for showing effective anti-inflammatory and analgesic properties and mainly used in osteoarthritis, rheumatoid arthritis, and ankylosing spondylitis.[2,3] Naproxen is rapidly and efficiently absorbed after oral administration but has a short half-life of 1-3 h and requires multiple dosing for maintaining therapeutic effect throughout the day.[4] The most frequent adverse side effects occurring with naproxen are gastrointestinal (GI) disturbances, peptic ulceration, and GI bleeding. To reduce the frequency of administrations and to improve patient compliance, naproxen is a suitable for making sustained release dosage form.[5,6]

Despite the advent of natural and synthetic polymers in microsphere preparation, there are number of advantages of lipid materials as matrixing agents like they are biocompatible, biodegradable, nonimmunogenic, can entrap wide range of water insoluble compounds and are economic as per as formulation of novel dosage form is concerned.[7] Preparation of controlled release lipid microspheres is commonly achieved using melt dispersion (congealable disperse phase encapsulation) technique, which utilizes fats of animals and/or vegetable origin as the matrix. This technique is especially suitable for drugs, which are insoluble in water. Taking into account the above considerations, lipid microspheres of naproxen using carnauba wax as a lipid carrier were prepared by modified melt dispersion technique.

## MATERIALS AND METHODS

### Materials

Naproxen was a gift sample from RPG Life Sciences Ltd. (Ankleshwar, India). Hydrogenated castor oil (HCO) was obtained from National Chemicals, Vadodara. Carnauba wax and glycerol monosterate were provided by Rankem (RFCL Limited), New Delhi, India. All other chemicals used were of analytical grade.

### Methods

#### Preparation of lipid microspheres

Naproxen microspheres were prepared by modified melt dispersion technique.[8] Carnauba wax and HCO (lipid) with or without modifier were melt on the heating mantle. Naproxen (drug) was dispersed in the molten lipid. Temperature of this fatty phase was maintained at about 10 °C above the melting point of lipid (i.e., at 90 °C). Then, 0.1 N HCl containing poly vinyl alcohol (PVA) 0.5%w/v as a dispersant was maintained at about 5°C above the melting point of lipid (85 °C). The fatty phase was poured into this solution while continuous stirring at an 800 rpm to form an o/w emulsion. The resultant emulsion was then agitated for 3 min while maintaining the temperature. Hardening of the oily internal phase and formation of microspheres were accomplished by pouring, twice the emulsion volume of ice-cold acidified water (4°C) into the beaker and stirring continuously for further 15 min in an ice bath. Microspheres obtained were filtered under vacuum and washed five times with phosphate buffer (pH 7.4) to remove the unentrapped drug and dried in room temperature for 24 h.

### Effect of formulation and process variables on evaluatory parameters[8]

The release of drug from microspheres is influenced by number of factors such as nature of carrier, drug concentration, dispersant concentration, stirring speed, stirring time, external-phase temperature, and external phase. Taking into account the above considerations, effects of various formulation and process variables on size, entrapment efficiency, and *in vitro* release of naproxen were studied. The different batches of microspheres were prepared by changing each of the parameters (one at a time) and keeping others constant.

#### Effect of drug-lipid ratio

The effect of drug-lipid ratio was studied on the preparation of naproxen-loaded microspheres using carnauba wax[9] and HCO as a carrier. Different batches with drug-lipid ratio of 1:3, 1:4, and 1:5 were prepared by modified melt dispersion technique as shown in Table 1. The other variables were kept constant. The effect of variables on microspheres prepared was then evaluated for different evaluatory parameters such as size, entrapment efficiency, and *in vitro* release.

**Table 1:** Effect of drug-lipid ratio on evaluatory parameters

Batches with carnauba wax	A1	A2	A3
Batches with hydrogenated castor oil	A4	A5	A6
Drug:lipid	1:3	1:4	1:5
Modifier	No	No	No
Dispersant	PVA (0.5% w/v)	PVA (0.5% w/v)	PVA (0.5% w/v)
Stirring speed	800 rpm	800 rpm	800 rpm
Stirring time	3 min	3 min	3 min

#### Effect of concentration of modifier

The effect of glycerol monosterate as a lipid modifier with different concentrations was studied in combination with carnauba wax by the same technique. Different batches with 25% w/w, 37.5% w/w, and 50% w/w of glycerol monostearate in combination with carnauba wax were prepared as shown in Table 2.

**Table 2:** Effect of modifier concentration on evaluatory parameters

Batch	B1 (25%w/w)	B2 (37.5%w/w)	B3 (50%w/w)
Mean diameter (μm) ± SD	182 ± 33	235 ± 45	292 ± 34
Entrapment efficiency (%)	53.73%	49.60%	37.44%

#### Effect of concentration of dispersant

The effect of various concentrations of PVA (dispersant) was studied by preparing different batches with 0.25% w/v, 0.5% w/v, and 0.75% w/v of PVA with respect to volume of dispersion media. All other variables were kept constant.

#### Effect of stirring speed

The effect of stirring speed on the preparation of microspheres was studied by preparing various batches with 500, 800, and 1100 rpm, using a variable speed mechanical stirrer and keeping all other variables constant.

#### Effect of stirring time

The effect of emulsification time was studied on the preparation of microspheres. The batches with varying emulsification-stirring time of 1, 3, and 5 min were prepared and evaluated, keeping all other variables constant.

#### Effect of temperature of external phase

The effect of temperature of external phase (0.1 N HCl) on the preparation of microspheres were studied at different temperature like ≤85°, 85, and ≥85 °C keeping all other variables constant. For all the batches, carnauba wax was used as a lipid material and 0.1 N HCl containing optimum concentration of PVA was used as a dispersion medium. The effect of variables on microspheres prepared was then evaluated for different evaluatory parameters such as size, entrapment efficiency, and *in vitro* release.

#### Effect of external phase

The effect of different external phase on the preparation of microspheres such as water, 0.1 N HCl, and pH 7.4 phosphate buffer was studied keeping all other variables constant.

### Evaluatory parameters

#### Size

Size distribution analysis of microspheres was done by optical microscopy using motic microscope. The diameters were sized using a suitable objective (10× and 40×). An average of 50 particles was calculated for each variable studied.[10]

#### Entrapment efficiency[9]

Entrapment efficiency in microspheres is very important to study the efficiency of the process. Entrapment efficiency of all the batches prepared to study the effect of various variables was determined spectrophotometrically using UV 2300, Techcomp. An approximate weight of about 100 mg of microspheres containing drug was added to phosphate buffer (pH 7.4) solution. The mixture was heated in order to melt lipid microspheres and to extract the drug into the solution. Then, this solution was allowed to cool up to room temperature to precipitate out the lipids and then filtered. After diluting with sufficient quantity of phosphate buffer (pH 7.4), drug concentration was determined by UV spectrophotometry at 317 nm using pH 7.4 phosphate buffer as a blank. Entrapment efficiency was calculated by using following formula:

$$\mathrm{Entrapment}\;\mathrm{efficiency}\;=\;\frac{Drug\;entrapped\;}{Theoretical\;drug\;content}\;\times \;100.$$

#### In vitro release studies[9]

United State Pharmacopoeia (USP) rotating basket method II (paddle type) has been employed for dissolution testing of microspheres at 50 rpm with temperature of 37 ± 0.5°C (Electrolab TDT-06P). Microspheres that showed acceptable physical properties were selected for the *in vitro* release studies. The procedure and composition of dissolution media were as per USP method described for naproxen tablets. Five-hundred milligrams of naproxen microspheres were placed into the dissolution apparatus. Five milliliters of dissolution medium was withdrawn by a pipette after a regular interval of 1, 2, 3, 4, 5, 6, 7, 8, 9, 10, 11, 12, and 24 h. The volume withdrawn was replaced with fresh quantities of dissolution fluid (phosphate buffer pH 7.4) maintained at 37 ± 0.5°C. Samples withdrawn were diluted to 10 mL with pH 7.4 phosphate buffer and filtered. The filtered samples were then analyzed at 317 nm against blank using double beam spectrophotometer, and absorbance was noted. The amount of drug released was calculated using the standard calibration curve for naproxen.

### Characterization of microspheres

#### Surface topography by SEM

SEM photographs were taken using scanning electron microscope model JOEL-LV-5600, USA at suitable magnification at room temperature. The shape and surface morphology of carnauba wax loaded naproxen microspheres were investigated using SEM. The samples for SEM study were prepared by lightly sprinkling the formulation on a double-adhesive tape stuck to an aluminum stub. The stubs were then coated with gold to a thickness of ~300 Å under an argon atmosphere using a gold sputter module in a high-vacuum evaporator. The coated samples were then randomly scanned and photomicrographs were taken.[11]

#### FT-IR spectroscopy

The FT-IR spectra were taken from dried samples. A FT-IR was used for the analysis in the frequency range between 4000 and 600 cm^-1^, an 8 cm^-1^ resolution and a 0.2 cm^-1^ rate (8400 S, Shimadzu Asia Pacific Pt. Ltd, Singapore). The samples (2 mg of the pure drug naproxen, empty microspheres, and naproxen-loaded carnauba wax microspheres) were selected separately and dispersed in KBr powder: the pellets were made by applying 6000 kg/cm^2^ and analyzed. Spectral measurements were obtained by powder diffuse reflectance on a FT-IR spectrophotometer.

#### Drug release kinetics

To study the exact mechanism of drug release from the microsphere, drug release data were analyzed according to zero-order, first-order, Higuchi square root, Hixson Crowell, and Peppas equation. The criteria for selecting the most appropriate model were chosen on the basis of goodness of fit test.[11]

## RESULTS AND DISCUSSION

**Figure 1:**
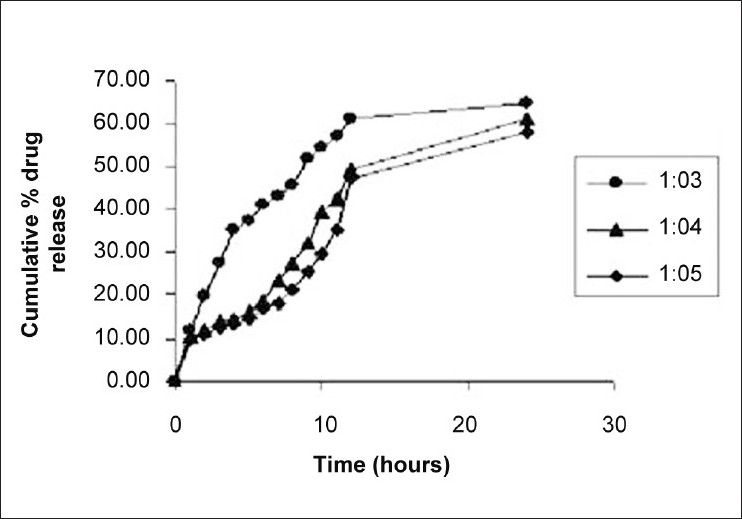
Effect of drug-lipid (carnauba wax) ratio on *in vitro* dissolution profile of naproxen in pH 7.4 phosphate buffer

**Figure 2:**
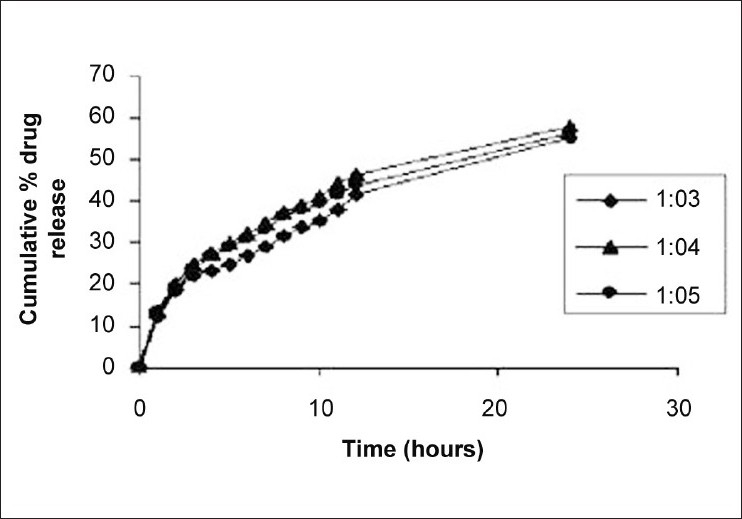
Effect of drug-lipid (hydrogenated castor oil) ratio on *in vitro* dissolution profile of naproxen

Effect of drug-lipid ratio revealed that entrapment efficiency value was high in case of carnauba wax and HCO, i.e., more than 80% up to drug-lipid ratio of 1:4, while it is drastically decreased in both the lipids, if the lipid ratio was increased to 1:5. This decrease in entrapment efficiency might be due to maximum solubility of the drug in the lipid melt and any further increase in lipid amount might lead to its expulsion out of the melt. The optimum drug-lipid ratio for the preparation of carnauba wax microspheres with high-entrapment efficiency (83.59%) and particle size (159 ± 33 μm) was found at the ratio of 1:3, while in case of HCO, the optimum drug-lipid ratio was also 1:3 with 89.87% entrapment efficiency and 169 ± 34 μm particle size. As lipid amount was increased, the mean diameter of microspheres in both the cases was increased. This might be due to increase in viscosity of lipid melted with drug, which consequently increased the diameter of emulsion droplets during emulsification and ultimately the mean diameter of microspheres. Effect of drug-lipid ratio on *in vitro* release profile of naproxen with carnauba wax as a carrier is shown in Figure 1. It was observed that the drug-lipid ratio 1:3 showed greater release, i.e., up to 70% in 24 h as compared to other because of finer particle size, thus increasing the surface area for dissolution. Also, in case of HCO as shown in Figure 2, it was observed that the drug-lipid ratio 1:3 shows greater release but less than that of carnauba wax (60% up to 24 h).

From the above-mentioned study, it could be revealed that the release rate of naproxen was greater from the lipid carnauba wax as compared to HCO. This might be due to low-melting point of carnauba wax than the HCO. Because the higher the melting point of lipid/wax, the slower was the cumulative amount of drug release. Thus, melting point of lipid/wax is inversely proportional to drug release. Therefore, carnauba wax was used in further studies.

**Figure 3:**
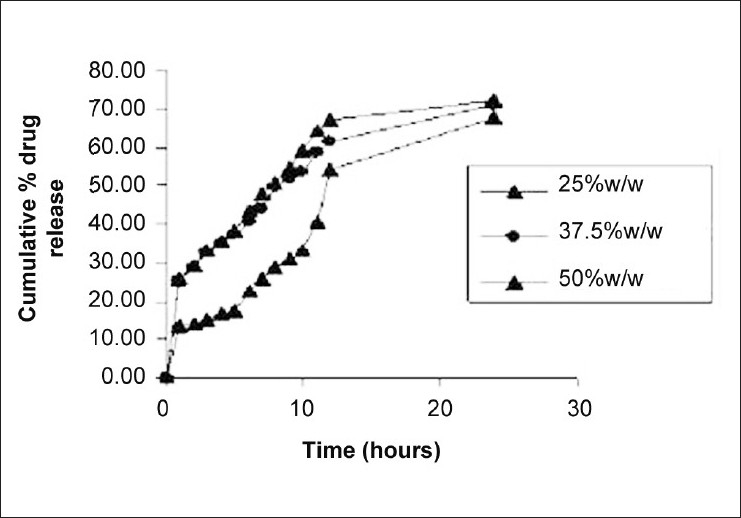
Effect of modifier (glycerol monosterate) concentration on *in vitro* dissolution profile of Naproxen

Effect of concentration of modifier shows low-entrapment efficiency with optimum particle size. However, as the concentration of modifier was increased, mean diameter was found to be increased, with a maximum of 292 ± 34 [(μm) ± SD] with 50% w/w concentration of glycerol monosterate. This might be due to increase in viscosity by the addition of modifier. Effect of modifier concentration on *in vitro* release profile of naproxen is shown in Figure 3. In vitro release studies showed that the release rate of the naproxen was increased by the addition of the modifier as compared to drug-lipid ratio. The reasons for greater release might be decrease in the melting temperature of lipid by the addition of the modifier. As the concentration of modifier was increased, drug release was found to be slower and lesser was the cumulative amount of drug released.

**Figure 4:**
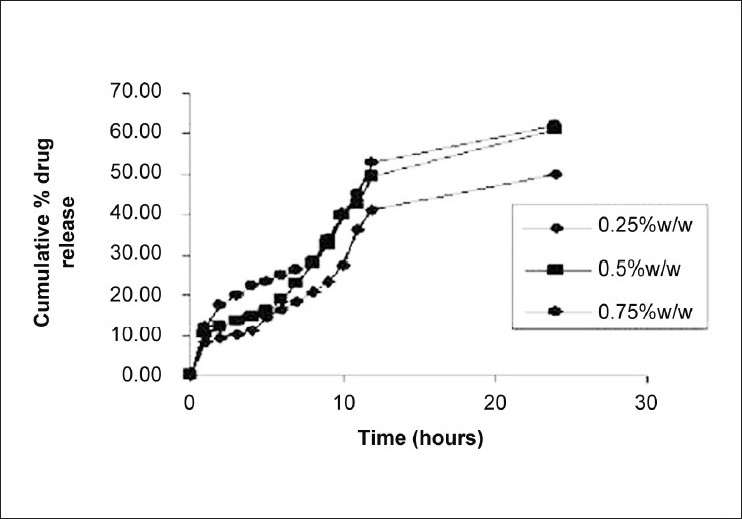
Effect of dispersant concentration on *in vitro* dissolution profile of naproxen

Effect of concentration of dispersant shows that, as the concentration of dispersant was increased, entrapment efficiency was increased. Use of polyvinyl alcohol (0.75% w/v) resulted in discrete, spherical uniform microspheres with 83.68% entrapment efficiency and 167 ± 39 particle size. Thus, polyvinyl alcohol in the concentration of 0.75% w/v was found to be optimum dispersant for microsphere preparation. As the concentration of dispersant was increased, mean diameter of microspheres was decreased. At low-dispersant concentrations, the droplets were poorly stabilized and tend to coalesce and became larger particle. Another reason for decrease in mean diameter might be decrease in interfacial tension between the hydrophobic material and aqueous external phase leading to formation of initially smaller droplets yielding smaller microspheres. Effect of dispersant concentration on *in vitro* release profile of naproxen is shown in Figure 4. From the results, it was revealed that batch C3 shows greater release profile of naproxen as compared to other two batches. Thus, the 0.75% w/v concentration of polyvinyl alcohol shows the greater release as compared to other because of finer particle size.

**Figure 5:**
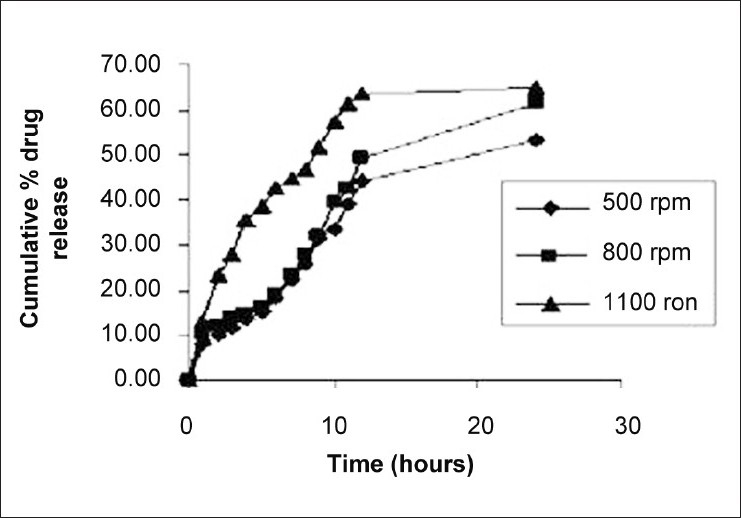
Effect of stirring speed on *in vitro* dissolution profile of Naproxen

Optimum stirring speed, which yielded microspheres with highest entrapment efficiency of 85.33% and particle size of 125 ± 58 μm was found to be 1100 rpm. Higher stirring speed (i.e., 1100 rpm) resulted in decrease in emulsion droplet size and higher proportion of smaller microspheres was formed as can be seen from mean diameter. On the other hand, stirring speed below 800 (i.e., 500 rpm) resulted in the formation of microspheres with larger mean diameter. Effect of stirring speed on *in vitro* release profile of naproxen is shown in Figure 5. It was observed that the batch D3 showed greater release of naproxen than that of D1 (500 rpm) and D2 (800 rpm). The reason for greater drug release from the batch D3 might be finer particle size, thus larger surface area of microspheres than that of microspheres prepared at 500 rpm (Batch D1) and 800 rpm (Batch D2).

**Figure 6:**
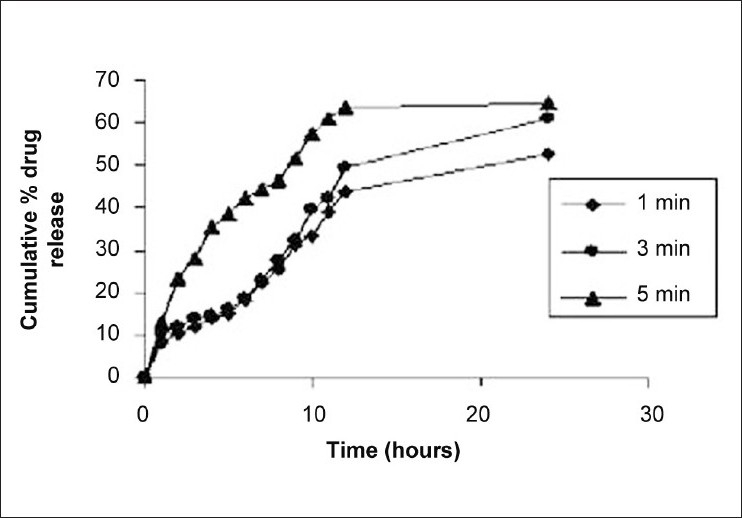
Effect of stirring time (emulsification) on *in vitro* dissolution profile of naproxen

Effect of stirring time revealed that emulsification-stirring time has also significant effect on entrapment efficiency and particle size of microspheres. However at an emulsification stirring time of 1 min (batch E1), agglomerates or aggregates were formed with large variation in mean diameter. Discrete, free flowing, uniform, and finer microspheres were formed at emulsification stirring time of 3 min (batch E2) and 5 min (batch E3). However, mean diameter of batch E2 was found to be greater than that of batch E3 (5 min). The reason for this increase in mean diameter followed by increasing emulsification time might be as follows; it has been reported that increase in homogenization time increases the energy input which might lead to decrease in the mean diameter of microspheres up to a minimum after which it might increase due to over emulsification. Effect of emulsification stirring time on *in vitro* release of naproxen is shown in Figure 6.

*In vitro* release profile of batches E1, E2, and E3 [Figure 6] showed that the drug release from batch E1 was slower than the other two batches. Again the reason for this slower drug release might be larger mean diameter of batch E1, leading to lesser surface area available for dissolution.

Effect of temperature of external phase revealed that the optimum temperature of external phase should be 85°C for the preparation of microspheres, i.e., 5°C greater than the melting point of wax (carnauba wax 80 ^°^C) and if it is greater or less than this temperature, it did not lead to the formation of microspheres.

Effect of external phase revealed that the microspheres were formed if the 0.1 N HCl was taken as the external phase. This might be due to the fact that the drug naproxen is soluble in pH 7.4 phosphate buffer which did not result in the formation of emulsion droplets while it is insoluble in water which also lead to the same condition and it is sparingly soluble in 0.1 N HCl which lead to the formation of emulsion droplets which after stirring formed the microspheres.

**Figure 7:**
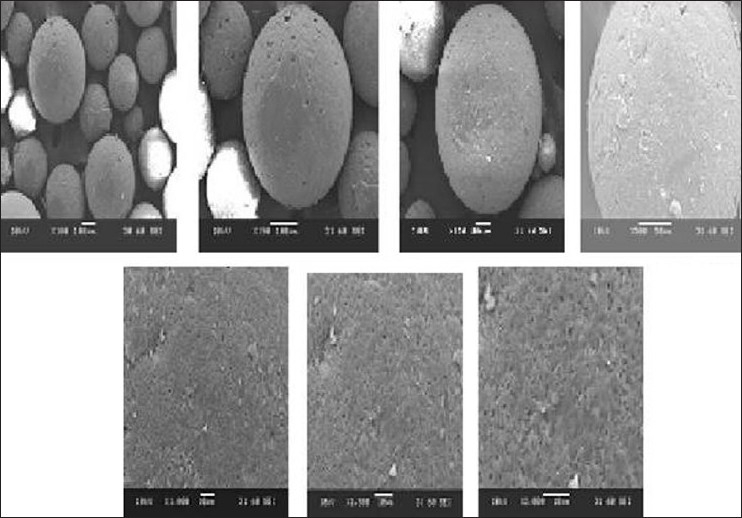
SEM photographs of microspheres (Batch A2) at ×100, ×110, ×190, ×500, ×1000, ×1500 and ×2000 magnification

Figure 7 shows SEM photographs of microspheres (Batch A2) at 100× magnification, 110×, 190×, 500×, 1000×, 1500×, and 2000× magnification. SEM photographs showed discrete, spherical, and uniform microspheres. SEM photographs also showed the absence of any drug crystal on the surface of microspheres revealing that the microspheres were smooth.

**Figure 8:**
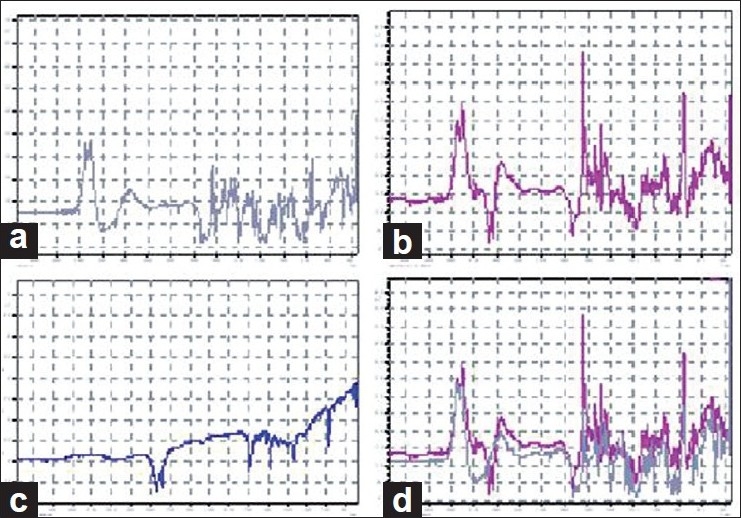
FT-IR Spectra of a) Naproxen b) Naproxen loaded microspheres c) Empty microspheres of carnauba wax d) Overlapping of and B

The FT-IR spectra of pure drug (naproxen), naproxen-loaded (carnauba) wax microspheres, empty microspheres, and overlapping of naproxen and naproxen-loaded microspheres are shown in Figure 8. From the FT-IR studies, it was observed that the peaks of naproxen were detected and identified in the spectrum of naproxen-loaded microspheres confirming that there was no drug-lipid interaction between naproxen and carnauba wax.

The mechanism of naproxen release from microspheres was studied by fitting the data obtained from *in vitro* release studies into zero-order, first order, matrix, Hixson-Crowell’s, and Peppas kinetic models. Obtained values of correlation coefficient are given in Table 3. It was found that all the batches showed better fitting with the Korsmeyer-Peppas model. The Korsmeyer-Peppas equation gave consistently higher values for the correlation coefficient (above 0.9545) than the other release models. Thus, the drug release from all the microsphere preparations confirmed Korsmeyer-Peppas model indicating the release mechanism may be by diffusion and erosion of the lipid matrix. This may be due to the fact that microspheres are matrix-type and swelling or erosion of microspheres took place during drug release experiments.

**Table 3:** Correlation coefficient values for release kinetics of lipid microspheres

Batch no.	Zero-order	First-order	Matrix (Higuchi)	Korsmeyer-Peppas	Hixson-Crowell
A1	0.5236	0.7938	0.9674	0.9746	0.7259
A2	0.9140	0.9332	0.9252	0.9610	0.9508
A3	0.9374	0.9247	0.9066	0.9610	0.9579
B1	0.2223	0.7939	0.9585	0.9698	0.6885
B2	0.2311	0.8148	0.9673	0.9786	0.7043
B3	0.9259	0.9270	0.9289	0.9679	0.9631
C1	0.9217	0.9428	0.9228	0.9545	0.9467
C3	0.8317	0.9397	0.9622	0.9632	0.9145
